# Gastric cancer and imaging biomarkers: Part 1 – a critical review of DW-MRI and CE-MDCT findings

**DOI:** 10.1007/s00330-018-5732-4

**Published:** 2018-10-02

**Authors:** Francesco Giganti, Lei Tang, Hideo Baba

**Affiliations:** 1 Department of Radiology, University College London Hospital NHS Foundation Trust, London, UK; 2 Division of Surgery and Interventional Science, Faculty of Medical Sciences, University College London, 3rd Floor, Charles Bell House, 43-45 Foley St, London, W1W 7TS UK; 3 Department of Radiology, Peking University Cancer Hospital, Beijing, China; 4 Department of Gastroenterological Surgery, Graduate School of Medical Sciences, Kumamoto University, Kumamoto, Japan

**Keywords:** Gastric cancer, Biomarkers, Magnetic resonance imaging, Tomography

## Abstract

**Abstract:**

The current standard of care for gastric cancer imaging includes heterogeneity in image acquisition techniques and qualitative image interpretation. In addition to qualitative assessment, several imaging techniques, including diffusion-weighted magnetic resonance imaging (DW-MRI), contrast-enhanced multidetector computed tomography (CE-MDCT), dynamic-contrast enhanced MRI and 18F-fluorodeoxyglucose positron emission tomography, can allow quantitative analysis. However, so far there is no consensus regarding the application of functional imaging in the management of gastric cancer. The aim of this article is to specifically review two promising biomarkers for gastric cancer with reasonable spatial resolution: the apparent diffusion coefficient (ADC) from DW-MRI and textural features from CE-MDCT. We searched MEDLINE/ PubMed for manuscripts published from inception to 6 February 2018. Initially, we searched for *(gastric cancer OR gastric tumour) AND diffusion weighted magnetic resonance imaging*. Then, we searched for *(gastric cancer OR gastric tumour) AND texture analysis AND computed tomography*. We collated the results from the studies related to this query. There is evidence that: (1) the ADC is a promising biomarker for the evaluation of the aggressiveness (T and N stage), treatment response and prognosis of gastric cancer; (2) textural features are related to the degree of differentiation, Lauren classification, treatment response and prognosis of gastric cancer. We conclude that these imaging biomarkers hold promise as effective additional tools in the diagnostic pathway of gastric cancer and may facilitate the multidisciplinary work between the radiologist and clinician, and across different institutions, to provide a greater biological understanding of gastric cancer.

**Key Points:**

*• Quantitative imaging is the extraction of quantifiable features from medical images for the assessment of normal or pathological conditions and represents a promising area for gastric cancer.*

*• Quantitative analysis from CE-MDCT and DW-MRI allows the extrapolation of multiple imaging biomarkers.*

*• ADC from DW-MRI and CE- MDCT-based texture features are non-invasive, quantitative imaging biomarkers that hold promise in the evaluation of the aggressiveness, treatment response and prognosis of gastric cancer.*

## Introduction

Gastric cancer is one of the most common malignancies worldwide [1].

The recommended management of this disease is a standardised multidisciplinary approach, which involves surgery, neoadjuvant and adjuvant chemotherapy. Surgical pathology is the reference standard for staging gastric cancer, and the Tumour, Node, Metastasis (TNM) classification is the most important tool for an adequate therapeutic plan and for predicting treatment response and prognosis [2]. Imaging plays a crucial role in the detection, staging, treatment planning and follow-up of this disease [3, 4].

A biomarker indicates a biological process (normal or pathological) both at baseline and after therapeutic interventions [5]. Oncological imaging represents an ideal setting for the collection of new biomarkers from different techniques, as oncological patients usually undergo multiple imaging studies [6]. The European Society of Radiology published a white paper on imaging biomarkers that underlined their importance as non-invasive, quantitative tools with different applications in oncology [7]. The Radiological Society of North America has also promoted the use of imaging biomarkers in clinical trials [8].

Quantitative imaging is the extraction of quantifiable features from medical images to assess normal or pathological conditions [9, 10].

There is a wide range of imaging biomarkers from different techniques. It is possible to extrapolate multiple imaging biomarkers through quantitative analysis of data derived from different techniques, such as contrast-enhanced multidetector computed tomography (CE-MDCT), diffusion-weighted magnetic resonance imaging (DW-MRI), dynamic contrast-enhanced (DCE)-MRI and 18F-fluorodeoxyglucose positron emission tomography (18F-FDG-PET) [11–19].

While reviews and meta-analyses of other imaging modalities (e.g. 18 FDG-PET) have been already carried out [20–25], this is the first time that a critical review has been specifically designed to summarise the state of the art of imaging biomarkers such as ADC from DW-MRI and texture features from CE-MDCT in gastric cancer.

Currently, there are only a few defined diagnostic and prognostic biomarkers for gastric tumours but none are related to imaging techniques [26–28].

In an attempt to overcome such limitations, in this review we specifically provide the state of the art on the use of two imaging biomarkers: the apparent diffusion coefficient (ADC) from DW-MRI and texture features from CE-MDCT.

## Evidence acquisition

**Fig. 1:**
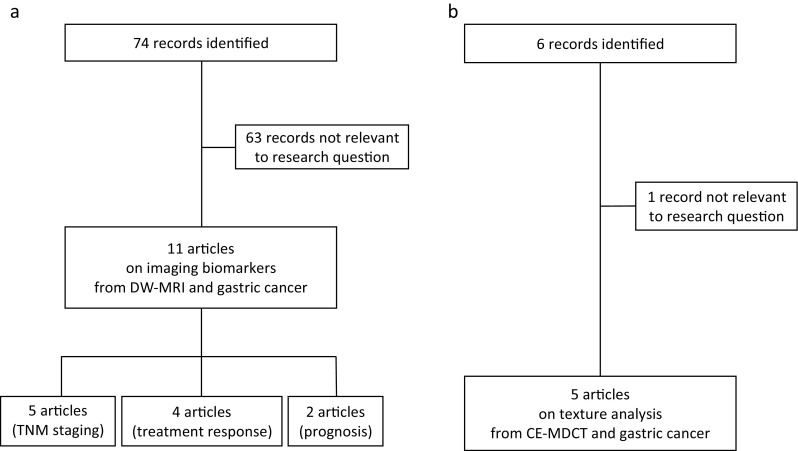
Flow diagrams showing the outcome of the initial searches resulting in the full studies included in the review for diffusion-weighted magnetic resonance imaging (DW-MRI) (**a**) and contrast-enhanced multidetector computed tomography (CE-MDCT) (**b**)

We searched MEDLINE/ PubMed for manuscripts published from inception to 6 February 2018. Initially we searched for *(gastric cancer OR gastric tumor) AND diffusion weighted magnetic resonance imaging.* Then, our search included *(gastric cancer OR gastric tumour) AND texture analysis AND computed tomography*. Figure 1 shows the literature search and study selection.

## DW-MRI and imaging biomarkers

Eleven studies on DW-MRI were relevant to our query, and they were assessed in full.

### Basic concepts of DW-MRI

DW-MRI measures the mobility of water molecules within tissues. Water diffusion is restricted in highly cellular tissues such as tumours, but some conditions (fibrosis/inflammation) can also show restricted diffusion [29].

**Fig. 2:**
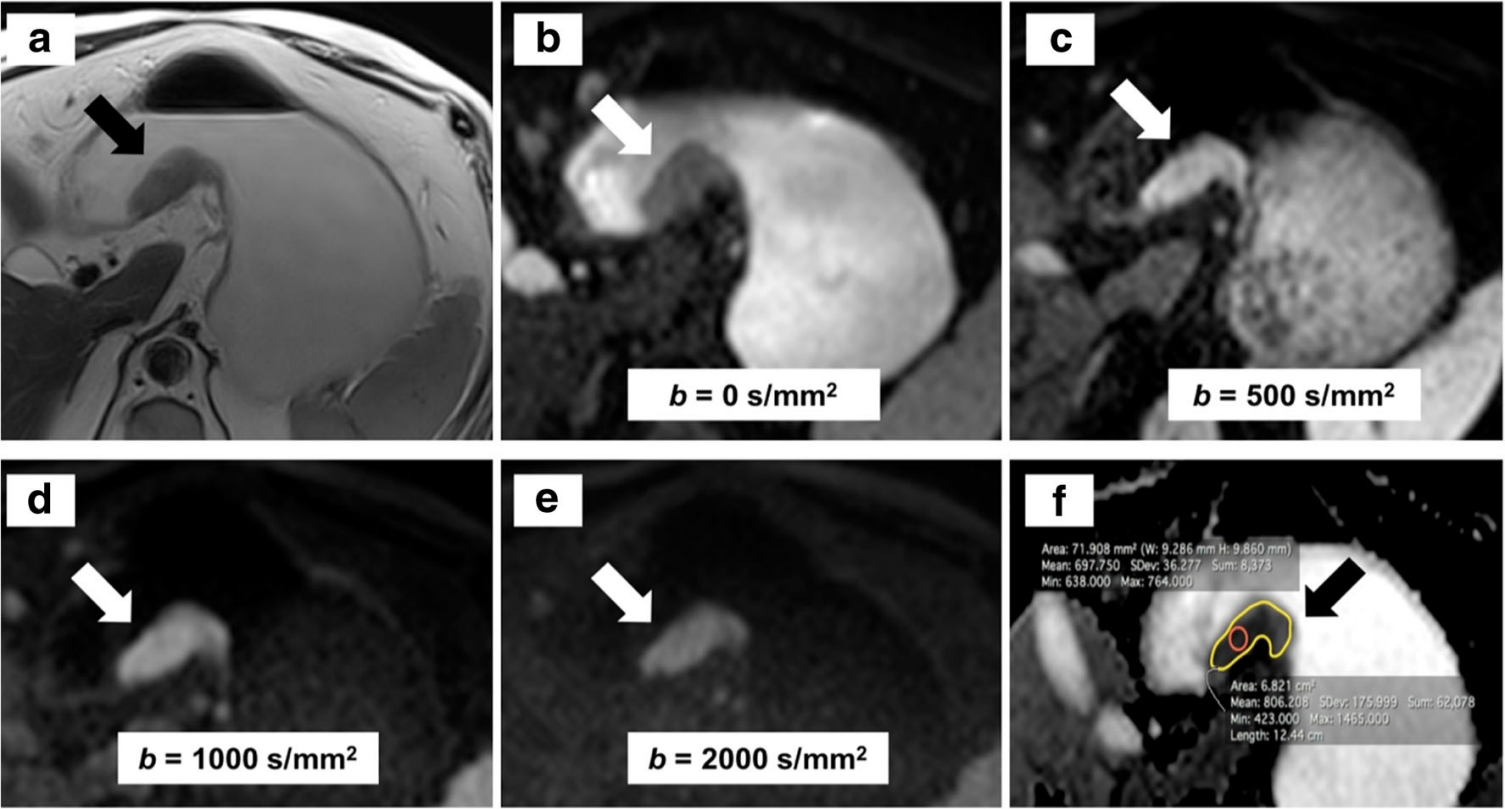
The arrows indicate a tumour of the lesser curvature on T2-weighted imaging (**a**) and on diffusion-weighted magnetic resonance imaging (DW-MRI) using different *b* values (**b**–**e**). The apparent diffusion coefficient (ADC) is calculated on the ADC map (**f**) from a small (red) or large (yellow) region of interest (red) on a single slice

Tissues with higher cellularity result in higher signal intensity on DWI. The *b* value is a parameter that identifies the measurement's sensitivity to water diffusion. The optimal *b* value should attenuate the healthy background tissue more than the lesion, with a reasonable signal-to-noise ratio at the same time. The greater the *b* value is, the stronger the DWI signal and the detection of pathological areas [29]. It is possible to generate a dedicated map that enables the calculation of the ADC. An area of higher signal intensity on DWI appears as a low-signal intensity on the ADC map. The region of interest (ROI) from which the ADC is calculated can be drawn as a small area or as a larger area encompassing the whole tumour on a single slice (Fig. 2). The ADC can also be obtained drawing the ROIs on all the slices where the tumour is visible (planimetry). This parameter is therefore a quantitative measurement of the mobility of water molecules within tissues and tumours, given their higher cellularity, have lower ADC values than normal areas [30].

### DW-MRI to assess the aggressiveness of gastric cancer

MRI of abdominal organs is often influenced by macroscopic motion. Imaging acquisition should be performed with breath-hold or respiratory triggering sequences, after the administration of intravenous/intramuscular anti-peristaltic agents and oral water to distend the gastric lumen [30, 31].

Growing evidence supports the use of DW-MRI in the assessment of the aggressiveness of gastric cancer [32–43]. Table 1 summarises the recent studies that have addressed this topic by analysing the role of ADC.

**Table 1:** DW-MRI and aggressiveness of gastric cancer

Study (ref.)	Year	Country	Type of study	No. of patients	MRI system	*b* values (s/mm^2^)	ROI placement for ADC calculation	ADC values (× 10^-3^ mm^2^/s)	Reference standard	Key message
Cheng et al [38]	2013	China	Prospective	28	1.5 T	0, 400, 800	Lymph nodes	1.55 vs. 1.28 ^a^	Radical surgery	ADC can predict metastatic lymph nodes
Liu et al [35]	2015	China	Retrospective	70	3.0 T	0, 1000	Primary tumour	1.01 vs. 0.89 ^b^ 1.25 vs. 0.97 ^a^	Radical surgery	ADC correlates with postoperative TNM stage and can be useful in assessing nodal involvement
Hasbahceci et al [42]	2015	Turkey	Prospective	23	1.5 T	50, 400, 800	Lymph nodes	1.02 vs. 0.90 ^a^	Radical surgery	No significant difference in ADC for nodal involvement
Zhong et al [41]	2016	China	Retrospective	82	3.0 T	0, 1000	Lymph nodes	1.48 vs. 1.15 ^a^	Radical surgery	The addition of DWI to CT and conventional MR scans yields higher accuracy for detecting metastatic lymph nodes
Giganti et al [37]	2017	Italy	Retrospective	89	1.5 T	0, 600	Primary tumour	1.57 vs. 1.22 ^c^ 1.81 vs. 1.30 ^a^	Radical surgery	ADC is significantly different according to local invasion and nodal involvement

*MRI* magnetic resonance imaging, *ROI* region of interest, *ADC* apparent diffusion coefficient, *TNM* Tumour, Node, Metastasis

^a^N0 vs. N+

^b^T3 vs. T4

^c^T1-3 vs. T4

Cheng et al [38] calculated ADC of pathological lymph nodes (dimensional criteria ≥ 5 mm in short-axis diameter) on a pixel-by-pixel base. After extended lymphadenectomy, metastatic lymph nodes showed lower median ADC than benign lymph nodes (1.28 vs. 1.55 × 10^-3^ mm^2^/s, *p* < 0.001). A cut-off of 1.39 × 10^-3^ mm^2^/s could discriminate between pathological and benign lymph nodes with a sensitivity of 85.7% and specificity of 79.4%. Quantitative results were also compared with morphological factors including size, border irregularity and enhancement patterns. The authors concluded that ADC holds promise to identify metastatic lymph nodes in gastric cancer. Their results are relevant, but lymph nodes < 5 mm were not evaluated because of the limited resolution of DWI.

Liu et al [35] conducted a retrospective study where the mean and minimum ADCs of gastric cancer were calculated and compared with surgical specimens. Both parameters showed a significant difference in detecting the different degrees of local invasion and nodal involvement. In particular, the correlation (*r*) with postoperative T staging was -0.464 (mean ADC) and -0.476 (minimum ADC) (*p* < 0.001), while the correlation (*r*) with postoperative N staging was -0.402 (mean ADC) and -0.397 (minimum ADC) (*p* = 0.001 and 0.002, respectively).

The same group conducted a whole-lesion ADC histogram analysis in 80 patients comparing the results with radical specimens [43]. Significant differences for all ADC parameters with respect to T and N stages were found. In particular, the ADC_10%_ provided the largest area under the curve (0.794) to identify nodal involvement.

Liu et al [34] investigated the role of whole-volume ADC-based entropy (a parameter describing tumour heterogeneity) in the preoperative assessment of gastric cancer. Entropy-related parameters from the ADC map significantly correlated with postoperative T, N and overall stage as well as vascular and perineural invasion.

Conversely, Hasbahceci et al [42] showed that it is not feasible to evaluate nodal involvement in gastric cancer by means of ADC. The cohort comprised 23 patients and ADC did not differentiate between benign and metastatic lymph nodes. However, the accuracy of DW-MRI in identifying metastatic lymph nodes in this study was 52.17%, 65.21% and 69.56% for group Ia, Ib and II, respectively. The overall accuracy of N-staging based on DW-MRI was 13%. The lack of motion correction manoeuvres and the small number of patients represent important limitations of this study.

Zhong and colleagues [41] showed that pathological lymph nodes have a lower ADC than benign lymph nodes (1.15 vs. 1.48 × 10^-3^ mm^2^/s, respectively; *p* <0.001). The same group compared the results from DWI with morphological MRI (T2-weighted imaging) and CE-MDCT; the combination of the three techniques yielded the highest area under the curve (0.893) for nodal involvement. The results are interesting, but a rigorous radiological-pathological correlation could not be assessed because of the large number and complex anatomic location of lymph nodes in gastric cancer. Another study [37] conducted on 89 patients reported that ADC is different according to local invasion, nodal involvement and the 7th TNM edition stage groups (*p* < 0.001). Therefore, there is evidence that ADC is a promising, non-invasive imaging biomarker for the evaluation of the aggressiveness of gastric cancer.

### DW-MRI in treatment response of gastric cancer

In recent years, there has been considerable interest in the application of DW-MRI to assess treatment response in gastric cancer [44–48]. Table 2 reports the main studies that have addressed this topic by means of ADC. Two studies from the same centre [46, 47] showed that ADC could be considered a reliable indicator of treatment response. De Cobelli et al [46] analysed ADC before and after treatment and compared the results with tumour volume, considering tumour regression grade (TRG) as the reference standard [49]. Responders showed a significant increase in ADC compared with non-responders (85.45% increase vs. 8.21% decrease, respectively: *p* < 0.001). However, this study also included patients with oesophageal cancer. Similar results [47] were found comparing ADC and standardised uptake volume (SUV) values in 17 patients undergoing DW-MRI and PET, before and after therapy. Changes in ADC were correlated with TRG (*r* = -0.78; *p* < 0.001), while SUV did not yield significant results. The results from this study are promising even though the number of patients is small (*n* = 17).

**Table 2:** DW-MRI and treatment response in gastric cancer

Study (ref.)	Year	Country	Type of study	No. of patients	MRI system	*b* values (s/mm^2^)	ROI placement for ADC calculation	Pre-treatment ADC in responders (× 10^-3^ mm^2^/s)	Pre-treatment ADC in non-responders (× 10^-3^ mm^2^/s)	Post-treatment ADC in responders (× 10^-3^ mm^2^/s)	Post-treatment ADC in non-responders (× 10^-3^ mm^2^/s)	Key message
De Cobelli et al [46]	2013	Italy	Prospective	23 ^a^	1.5 T	0, 600	Primary tumour	1.32	1.63	2.22	1.51	ADC changes can be considered a reliable indicator of treatment response
Giganti et al [47]	2014	Italy	Prospective	17	1.5 T	0, 600	Primary tumour	1.39	1.67	1.89	1.38	Post-treatment ADC is a good marker of treatment response
Lee et al [44]	2015	Korea	Prospective	11	3.0 T	100, 500, 1000	Primary tumour	1.08 ^b^	1.16 ^b^	NR	NR	ADC is not significantly different between responders and non-responders
Zhong et al [45]	2016	China	Prospective	106	3.0 T	0, 1000	Lymph nodes	1.11 ^c^ 1.15 ^d^	1.19 ^e^	NR 1.64 ^d^	1.53 ^e^	ADC can predict nodal response to chemotherapy

*MRI* magnetic resonance imaging, *ROI* region of interest, *ADC* apparent diffusion coefficient, *NR* not reported

^a^Subgroup considering Siewert III and gastric cancer

^b^Averaged between two readers

^c^Complete response

^d^Partial response

^e^Stable disease

Lee and colleagues [44] investigated the usefulness of PET/MRI in predicting treatment response for advanced, unresectable gastric cancer in 11 patients. In addition to ADC, SUV and perfusion parameters were calculated prior to chemotherapy. Response to treatment was evaluated on the basis of the response evaluation criteria in solid tumours (RECIST) 1.1 criteria [50]. Differently from perfusion parameters, ADC and SUV were not significantly different between responders and non-responders. However, the lack of both post-treatment imaging and comparison with surgical specimens represents an important limitation of this study.

**Fig. 3:**
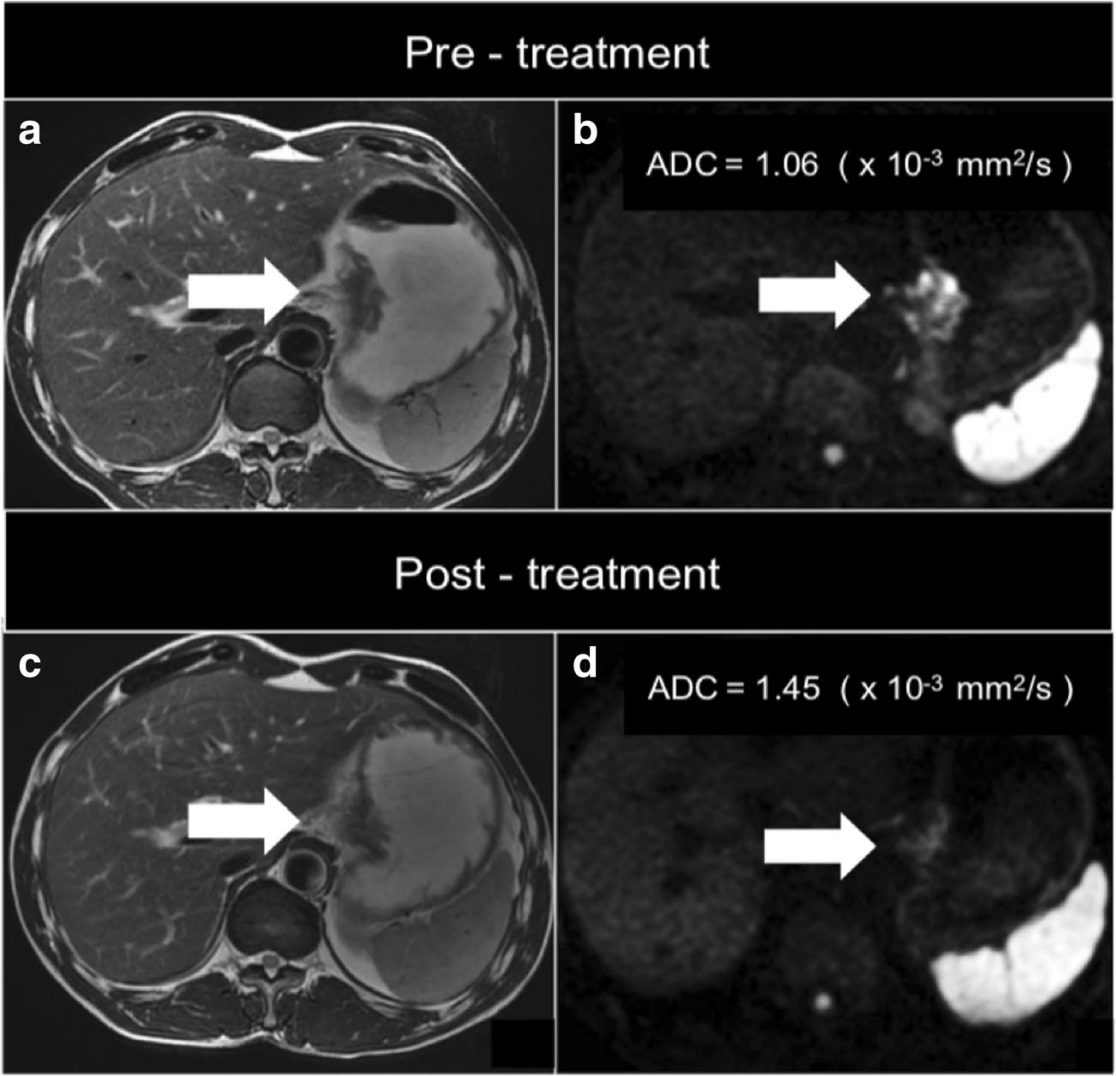
Image of a tumour of the gastro-oesophageal junction on T2-weighted (**a** and **c**) and diffusion-weighted (**b** and **d**) imaging before and after treatment. There has been a decrease in the conspicuity on DW-MRI (**d**) and an increase in the apparent diffusion coefficient (ADC) value after treatment (**d**)

Zhong and colleagues [45] conducted a study on 106 patients with biopsy-proven gastric cancer undergoing DW-MRI before and after chemotherapy at different time points. Mean ADC was calculated for benign and metastatic lymph nodes, and response to treatment was evaluated on the basis of the RECIST 1.1 criteria [50]. ADC significantly increased after treatment in all groups (complete response, partial response and stable disease group), predicting nodal response to neoadjuvant therapy. These studies support the potential role of DW-MRI in tailoring the therapeutic plan for gastric cancer (Fig. 3).

### DW-MRI in the prognosis of gastric cancer

At present, two studies [37, 51] have shown that ADC can be considered a prognostic biomarker for overall survival and risk stratification of gastric cancer, according to the 7th TNM edition [52]. The first study [37] reports that an ADC of 1.8 × 10^-3^ mm^2^/s may discriminate between stage I and stage II and that values ≤ 1.36 × 10^-3^ mm^2^/s are associated with stage III. The second paper [51] shows that an ADC ≤ 1.5 × 10^-3^ mm^2^/s is related to a negative outcome, along with T and N stages after surgery. These results confirm the growing interest that quantitative MR imaging in gastric cancer is gaining in the scientific community [53].

## CE-MDCT and imaging biomarkers

Five studies on CE-MDCT were relevant, and they were assessed in full (Fig. 1).

### Basic concepts of texture analysis

Texture analysis provides information on the intrinsic variation of pixels within an image. In oncology, this represents a non-invasive method to assess the heterogeneity within a tumour and could reflect its intrinsic aggressive biology or treatment resistance [54, 55]. There are different levels of textural features; these range from first-order statistics (that evaluate the grey-level distribution from pixels in a given area, such as energy, entropy, skewness) to higher-order statistics that investigate the relationship between different voxels [56].

This is a hot topic for quantitative imaging in oncology in order to evaluate lesion characterisation, response to therapy and prognosis [56]. However, there is still marked variability in methods and post-processing techniques in addition to the need to identify the key parameters among hundreds of potential imaging biomarkers from CE-MDCT [56].

### A standardised CE-MDCT protocol for adequate texture analysis in gastric cancer

An adequate CE-MDCT scan is crucial to obtain a reliable and reproducible texture analysis for research purposes. As a rule of thumb, the CE-MDCT scan parameters usually applied for gastric cancer are: 120 kVp, 250 mAS, collimation 64 × 0.625 mm, 3 mm helical thickness and 1 mm reconstruction thickness [57]. The first step for an adequate set of images is to ensure a correct distension of the gastric cavity. This can be reached by using negative endoluminal contrast agents (500–750 ml water or gas produced by effervescent granules).

**Fig. 4:**
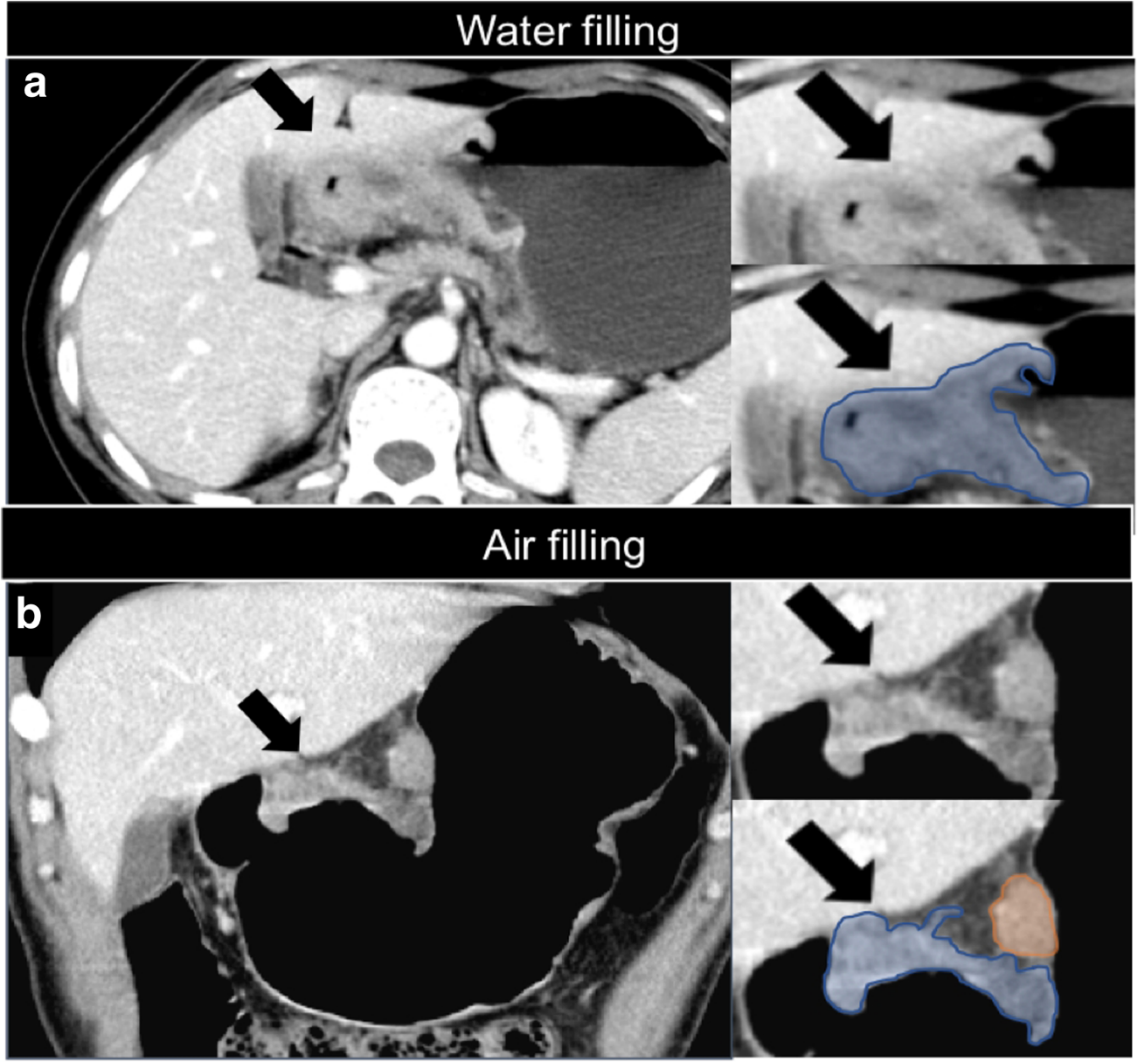
Tumour of the antrum (arrows) using water (**a**; axial view) and air filling (**b**; coronal view) on contrast-enhanced multidetector computed tomography. Air filling is more accurate when drawing the regions of interest (ROIs) for texture analysis (blue: primary tumour; orange: pathological lymph node)

Air filling is more accurate than water filling for tumours of the antrum; this is very important when drawing the regions of interest (ROIs) for texture analysis, as shown in Fig. 4.

**Fig. 5:**
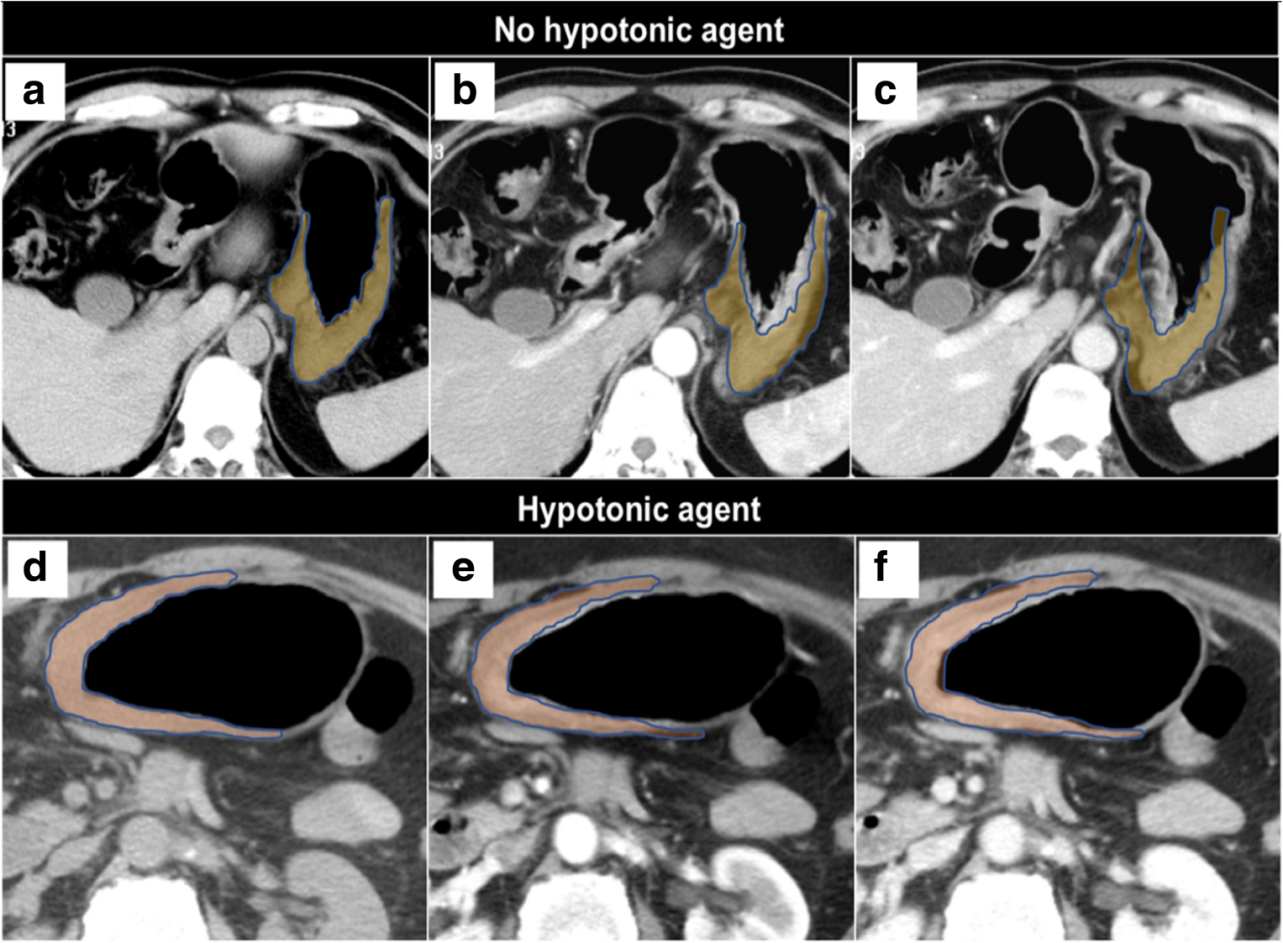
Comparison between a protocol without and with administration of anti-peristaltic agent on multidetector computed tomography. The image shows how the region of interest (ROI) delineation is more reproducible at different time points in the second scenario. Unenhanced (**a** and **d**), arterial (**b** and **e**) and portal (**c** and **f**) phases

**Fig. 6:**
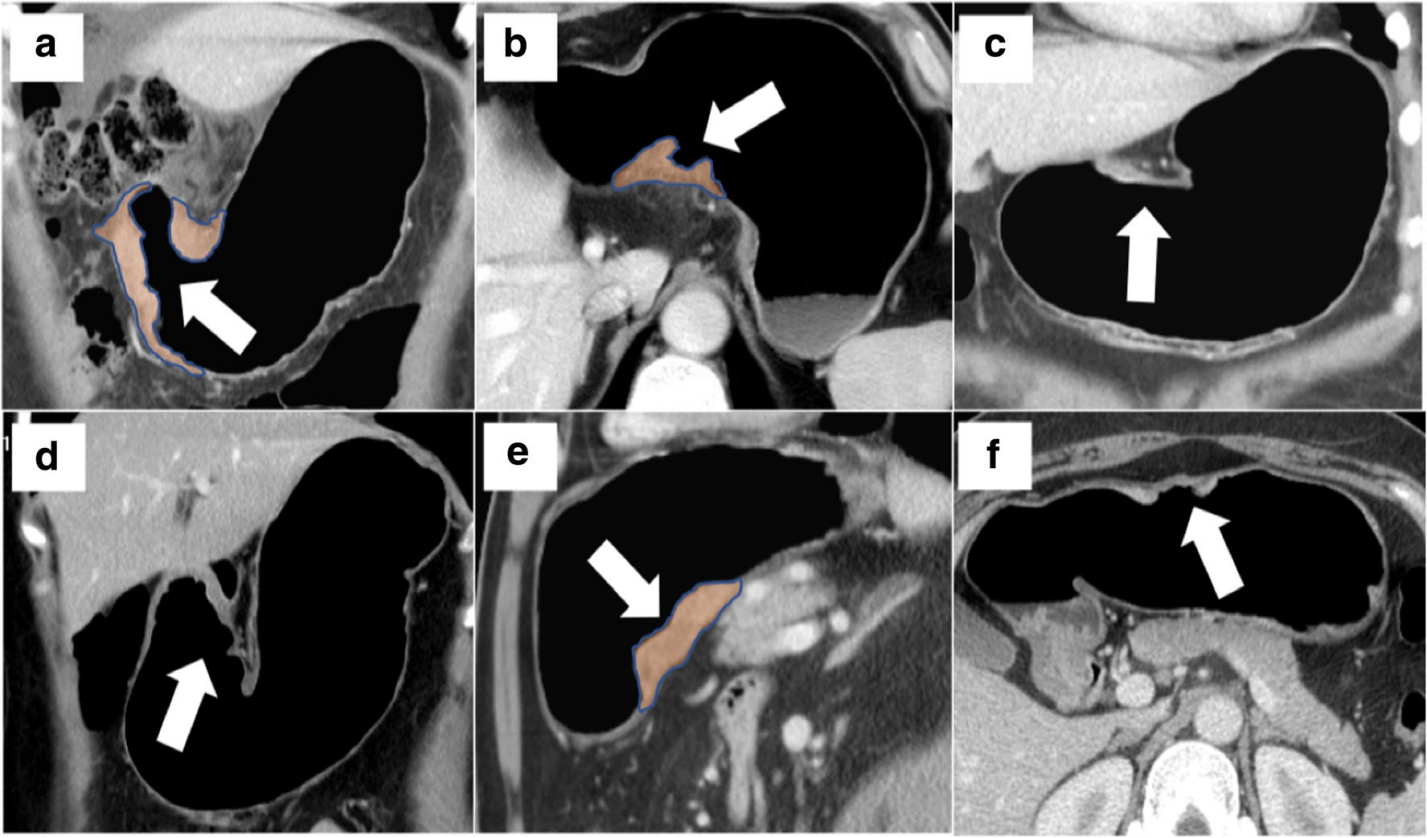
Contrast-enhanced multidetector computed tomography images of different gastric cancers (arrows) that have been acquired using a standard protocol, thus allowing accurate regions of interest for texture analysis (**a**, **b**, **e**, taken as an example)

The second step is the use of an intravenous/intramuscular anti-peristaltic agent (e.g. scopolamine-N-butyl bromide or glucagone) similarly to DW-MRI. This reduces the mobility of the gastric walls during the scan acquisition so that the ROIs can be drawn on static images and easily compared for texture analysis (Fig. 5). In this regard, the 8th TNM Staging Manual mentions the important role of gastric distention for the staging of gastric cancer [58]. Figure 6 is a set of different CE-MDCT images of gastric cancer acquired using the aforementioned recommendations, allowing accurate ROI delineations for texture analysis.

### Texture analysis and gastric cancer

Texture analysis has been receiving much attention in the management of gastric cancer. However, only a few studies have been published on this topic so far [59–63], and they are listed in Table 3.

**Table 3:** Texture analysis and gastric cancer

Study (ref.)	Year	Country	Type of study	No. of patients	ROI selection	CE-MDCT phase	Reference standard	Texture features	Key message
Ba-Ssalamah et al [59]	2013	Austria	Retrospective	47	Single slice	Arterial; venous	Biopsy	2nd order statistics	Texture analysis can differentiate histological subtypes
Giganti et al [62]	2016	Italy	Retrospective	56	Whole tumour	Late arterial	Radical resection	1st and 2nd order statistics	Texture analysis reflects tumour aggressiveness and is related to prognosis
Yoon et al [63]	2016	Korea	Retrospective	26	Single slice	Venous	Biopsy	2nd order statistics	Texture features are associated with better survival in HER2-positive patients receiving trastuzumab
Giganti et al [61]	2017	Italy	Retrospective	34	Whole tumour	Late arterial	Radical resection	1st and 2nd order statistics	Texture analysis provides important information on the response rate to neoadjuvant therapy
Liu et al [60]	2017	China	Retrospective	107	Single slice	Arterial; venous	Radical resection	1st and 2nd order statistics	Texture parameters can predict the degree of differentiation, Lauren classification and vascular invasion

*ROI* region of interest, *CE-MDCT* contrast-enhanced multidetector computed tomography, *HER* human epidermal growth factor receptor

First, there are no specific recommendations regarding the most accurate CE-MDCT phase for texture analysis. Some studies in Table 3 were conducted in the (late) arterial phase [61, 62], while others in the venous phase [63] or in both [59, 60].

However, the administration of intravenous contrast is important for texture analysis, as this allows the quantification of the vascularity before and after treatment and helps assessing the degree of infiltration of the surrounding fat. Texture analysis reflects some tumour features related to tissue heterogeneity at a cellular level; this means that differences at a microscopic level are also detectable macroscopically by CE-MDCT imaging [56].

A closer look at the results in Table 3 reveals that texture analysis can differentiate histological subtypes (adenocarcinoma, lymphoma and gastrointestinal stromal tumours) [59]. Similar results have been published by Liu et al [60]; the authors found that texture parameters can predict the degree of differentiation, Lauren classification and vascular invasion. There is also evidence that textural features can predict treatment response [61] when the response rate is based on TRG [49]. Like DW-MRI, quantitative imaging from texture analysis is associated with the prognosis of untreated [62] and treated [63] gastric cancer. The studies presented in Table 3 offer compelling evidence that CE-MDCT-based texture analysis holds promise in the management of gastric cancer, even though further studies are necessary before widespread application of this technique in common clinical practice.

## Limitations

Results so far have been encouraging but have given rise to many questions in need of further investigation as they limit the application of imaging biomarkers in daily clinical practice. First, the large variability in the selection of imaging acquisition parameters for DW-MRI and CE-MDCT (e.g. standardised *b* values, arterial vs. venous phase) is a considerable challenge. Future studies should aim at standardising data-acquisition protocols as well as validating new methods to extract quantitative parameters from imaging techniques (e.g. ROI assessment).

Second, multicentre trials on large cohorts of patients have yet to be carried out. Focused groups of researchers willing to perform multicentre, international studies are needed to address the key questions related to imaging biomarkers for gastric cancer [64]. The radiological community should continue to play an active role in the academic research by providing valuable expertise to incorporate imaging biomarkers into future clinical trials.

## Conclusions

As emerged from this paper, imaging biomarkers are promising tools of modern radiology [65, 66] and could become an essential cornerstone for gastric cancer therapy. Despite these limitations, this review highlights the great potential of ADC and texture analysis for gastric cancer. This is a vital issue for future guidelines, especially in the era of “personalised medicine”, where the goal is to tailor specific treatments for each single patient.

## Abbreviations

18F-FDG-PET: 18F-fluorodeoxyglucose positron emission tomography
ADC: Apparent diffusion coefficient
CE-MDCT: Contrast-enhanced multidetector computed tomography
DW-MRI: Diffusion-weighted magnetic resonance imaging
ROI: Region of interest
SUV: Standardised uptake volume
TNM: Tumour, Node, Metastasis
TRG: Tumour regression grade

## References

[1] Jemal A, Bray F, Center MM, Ferlay J, Ward E, Forman D (2011). Global cancer statistics. CA Cancer J Clin.

[2] Amin MB, Edge S, Greene F (2017). AJCC cancer staging manual.

[3] Giganti F, Orsenigo E, Arcidiacono PG (2016). Preoperative locoregional staging of gastric cancer: is there a place for magnetic resonance imaging? Prospective comparison with EUS and multidetector computed tomography. Gastric Cancer.

[4] Richman DM, Tirumani SH, Hornick JL (2017). Beyond gastric adenocarcinoma: multimodality assessment of common and uncommon gastric neoplasms. Abdom Radiol (NY).

[5] Biomarkers Definitions Working Group (2001). Biomarkers and surrogate endpoints: preferred definitions and conceptual framework. Clin Pharmacol Ther.

[6] O’Connor JP, Aboagye EO, Adams JE (2017). Imaging biomarker roadmap for cancer studies. Nat Rev Clin Oncol.

[7] European Society of Radioloy (ESR) (2010). White paper on imaging biomarkers. Insights Imaging.

[8] Buckler AJ, Bresolin L, Dunnick NR, Sullivan DC, Group (2011). A collaborative enterprise for multi-stakeholder participation in the advancement of quantitative imaging. Radiology.

[9] Obuchowski NA, Reeves AP, Huang EP (2015). Quantitative imaging biomarkers: a review of statistical methods for computer algorithm comparisons. Stat Methods Med Res.

[10] Kurland BF, Gerstner ER, Mountz JM (2012). Promise and pitfalls of quantitative imaging in oncology clinical trials. Magn Reson Imaging.

[11] European Society of Radiology (ESR) (2015). ESR position paper on imaging biobanks. Insights Imaging.

[12] European Society of Radiology (ESR) (2013). ESR statement on the stepwise development of imaging biomarkers. Insights Imaging.

[13] Van der Meel R, Gallagher WM, Oliveira S, O’Connor AE, Schiffelers RM, Byrne AT (2010). Recent advances in molecular imaging biomarkers in cancer: application of bench to bedside technologies. Drug Discov Today.

[14] Yankeelov TE, Abramson RG, Quarles CC (2014). Quantitative multimodality imaging in cancer research and therapy. Nat Rev Clin Oncol.

[15] Glunde K, Pathak AP, Bhujwalla ZM (2007). Molecular-functional imaging of cancer: to image and imaging. Trends Mol Med.

[16] Li HH, Zhu H, Yue L (2017). Feasibility of free-breathing dynamic contrast-enhanced MRI of gastric cancer using a golden-angle radial stack-of-stars VIBE sequence: comparison with the conventional contrast enhanced breath-hold 3D VIBE sequence. Eur Radiol.

[17] Ma L, Xu X, Zhang M (2017). Dynamic contrast-enhanced MRI of gastric cancer: Correlations of the pharmacokinetic parameters with histological type, Lauren classification, and angiogenesis. Magn Reson Imaging.

[18] Jang KM, Kim SH, Lee SJ, Lee MW, Choi D, Kim KM (2014). Upper abdominal gadoxetic acid-enhanced and diffusion-weighted MRI for the detection of gastric cancer: Comparison with two-dimensional multidetector row CT. Clin Radiol.

[19] Kang BC, Kim JH, Kim KW (2000). Value of the dynamic and delayed MR sequence with Gd-DTPA in the T-staging of stomach cancer: correlation with the histopathology. Abdom Imaging.

[20] Hallinan JT, Venkatesh SK (2013). Gastric carcinoma: imaging diagnosis, staging and assessment of treatment response. Cancer Imaging.

[21] Yoon H, Lee DH (2014). New approaches to gastric cancer staging: beyond endoscopic ultrasound, computed tomography and positron emission tomography. World J Gastroenterol.

[22] Wu L, Hu J, Hua J, Gu H, Zhu J, Xu J (2012) 18F-fluorodeoxyglucose positron emission tomography to evaluate recurrent gastric cancer: a systematic review and meta-analysis. J Gastroenterol Hepatol 27(3):472–480 10.1111/j.1440-1746.2011.06919.x 21916986

[23] Li P, Liu Q, Wang C (2016). Fluorine-18-fluorodeoxyglucose positron emission tomography to evaluate recurrent gastric cancer after surgical resection: a systematic review and meta-analysis. Ann Nucl Med.

[24] Zou H, Zhao Y (2013). 18FDG PET-CT for detecting gastric cancer recurrence after surgical resection: a meta-analysis. Surg Oncol.

[25] Hassanzadeh-Rad A, Yousefifard M, Katal S (2016). The value of (18) F-fluorodeoxyglucose positron emission tomography for prediction of treatment response in gastrointestinal stromal tumors: a systematic review and meta-analysis. J Gastroenterol Hepatol.

[26] Elimova E, Wadhwa R, Shiozaki H (2015). Molecular biomarkers in gastric cancer. J Natl Compr Canc Netw.

[27] Mi L, Ji X, Ji J (2016). Prognostic biomarker in advanced gastric cancer. Transl Gastrointest Cancer.

[28] Pinheiro Ddo R, Ferreira WA, Barros MB, Araújo MD, Rodrigues-Antunes S, Borges Bdo N (2014). Perspectives on new biomarkers in gastric cancer: diagnostic and prognostic applications. World J Gastroenterol.

[29] Koh DM, Collins DJ (2007). Diffusion-weighted MRI in the body: applications and challenges in oncology. AJR Am J Roentgenol.

[30] Padhani AR, Liu G, Koh DM, Chenevert TL (2009). Diffusion-weighted magnetic resonance imaging as a cancer biomarker: consensus and recommendations. Neoplasia.

[31] Sheybani A, Menias CO, Luna A (2015). MRI of the stomach: a pictorial review with a focus on oncological applications and gastric motility. Abdom Imaging.

[32] Luo M, Song H, Liu G (2017). Comparison of DWI and 18F-FDG PET/CT for assessing preoperative N-staging in gastric cancer: evidence from a meta-analysis. Oncotarget.

[33] Arslan H, Fatih Özbay M, Çallı İ (2017). Contribution of diffusion weighted MRI to diagnosis and staging in gastric tumors and comparison with multi-detector computed tomography. Radiol Oncol.

[34] Liu S, Zheng H, Zhang Y (2018). Whole-volume apparent diffusion coefficient-based entropy parameters for assessment of gastric cancer aggressiveness. J Magn Reson Imaging.

[35] Liu S, Wang H, Guan W (2015). Preoperative apparent diffusion coefficient value of gastric cancer by diffusion-weighted imaging: correlations with postoperative TNM staging. J Magn Reson Imaging.

[36] Liu S, He J, Guan W (2014). Added value of diffusion weighted MR imaging to T2-weighted and dynamic contrast-enhanced MR imaging in T staging of gastric cancer. Clin Imaging.

[37] Giganti F, Ambrosi A, Chiari D (2017). Apparent diffusion coefficient by diffusion-weighted magnetic resonance imaging as a sole biomarker for staging and prognosis of gastric cancer. Chin J Cancer Res.

[38] Cheng J, Wang Y, Deng J (2013). Discrimination of metastatic lymph nodes in patients with gastric carcinoma using diffusion-weighted imaging. J Magn Reson Imaging.

[39] Liu S, He J, Guan W, Li Q, Zhang X, Mao H (2014). Preoperative T staging of gastric cancer: comparison of diffusion- and T2-weighted magnetic resonance imaging. J Comput Assist Tomogr.

[40] Joo I, Lee JM, Kim H, Shin CI, Han JK, Choi BI (2015). Prospective comparison of 3T MRI with diffusion-weighted imaging and MDCT for the preoperative TNM staging of gastric cancer. J Magn Reson Imaging.

[41] Zhong L, Zhao W, Ren F (2016). Lymph node metastasis in patients with gastric cancer: a multi-modality, morphologic and functional imaging study. Am J Transl Res.

[42] Hasbahceci M, Akcakaya A, Memmi N (2015). Diffusion MRI on lymph node staging of gastric adenocarcinoma. Quant Imaging Med Surg.

[43] Liu S, Zhang Y, Chen L (2017). Whole-lesion apparent diffusion coefficient histogram analysis: significance in T and N staging of gastric cancers. BMC Cancer.

[44] Lee DH, Kim SH, Im SA, Oh DY, Kim TY, Han JK (2016). Multiparametric fully-integrated 18-FDG PET/MRI of advanced gastric cancer for prediction of chemotherapy response: a preliminary study. Eur Radiol.

[45] Zhong J, Zhao W, Ma W (2016). DWI as a quantitative biomarker in predicting chemotherapeutic efficacy at multitime points on gastric cancer lymph nodes metastases. Medicine (Baltimore).

[46] De Cobelli F, Giganti F, Orsenigo E (2013). Apparent diffusion coefficient modifications in assessing gastro-oesophageal cancer response to neoadjuvant treatment: comparison with tumour regression grade at histology. Eur Radiol.

[47] Giganti F, De Cobelli F, Canevari C (2014). Response to chemotherapy in gastric adenocarcinoma with diffusion-weighted MRI and (18) F-FDG-PET/CT: correlation of apparent diffusion coefficient and partial volume corrected standardized uptake value with histological tumor regression grade. J Magn Reson Imaging.

[48] Consolino L, Longo DL, Sciortino M (2017). Assessing tumor vascularization as a potential biomarker of imatinib resistance in gastrointestinal stromal tumors by dynamic contrast-enhanced magnetic resonance imaging. Gastric Cancer.

[49] Blackham AU, Greenleaf E, Yamamoto M (2016). Tumor regression grade in gastric cancer: predictors and impact on outcome. J Surg Oncol.

[50] Eisenhauer EA, Therasse P, Bogaerts J (2009). New response evaluation criteria in solid tumors: revised RECIST guideline (version 1.1). Eur J Cancer.

[51] Giganti F, Orsenigo E, Esposito A (2015). Prognostic role of diffusion-weighted MR imaging for resectable gastric cancer. Radiology.

[52] Edge SB, Byrd DR, Compton CC, Fritz AG, Greene F, Trotti A (2010). AJCC Cancer Staging Manual.

[53] Mills AF, Sakai O, Anderson SW, Jara H (2017). Principles of quantitative MR imaging with illustrated review of applicable modular pulse diagrams. Radiographics.

[54] Ganeshan B, Miles KA (2013). Quantifying tumour heterogeneity with CT. Cancer Imaging.

[55] Davnall F, Yip CS, Ljungqvist G (2012). Assessment of tumor heterogeneity: an emerging imaging tool for clinical practice?. Insights Imaging.

[56] Lubner MG, Smith AD, Sandrasegaran K, Sahani DV, Pickhardt PJ (2017). CT texture analysis: definitions, applications, biologic correlates, and challenges. Radiographics.

[57] Hallinan JT, Venkatesh SK (2013). Gastric carcinoma: imaging diagnosis, staging and assessment of treatment response. Cancer Imaging.

[58] Amin MB, Edge S, Greene F (2016). Stomach - Chapter 17. American Joint Committee on Cancer (AJCC) Cancer Staging Manual.

[59] Ba-Ssalamah A, Muin D, Schernthaner R (2013). Texture-based classification of different gastric tumors at contrast-enhanced CT. Eur J Radiol.

[60] Liu S, Liu S, Ji C (2017). Application of CT texture analysis in predicting histopathological characteristics of gastric cancers. Eur Radiol.

[61] Giganti F, Marra P, Ambrosi A (2017). Pre-treatment MDCT-based texture analysis for therapy response prediction in gastric cancer: Comparison with tumour regression grade at final histology. Eur J Radiol.

[62] Giganti F, Antunes S, Salerno A (2017). Gastric cancer: texture analysis from multidetector computed tomography as a potential preoperative prognostic biomarker. Eur Radiol.

[63] Yoon SH, Kim YH, Lee YJ (2016). Tumor heterogeneity in human epidermal growth factor receptor 2 (HER2)-positive advanced gastric cancer assessed by CT texture analysis: association with survival after trastuzumab treatment. PLoS One.

[64] deSouza NM, Winfield JM, Waterton JC (2018). Implementing diffusion-weighted MRI for body imaging in prospective multicentre trials: current considerations and future perspectives. Eur Radiol.

[65] Waterton JC, McShane LM, O’Connor JPB (2017). Imaging biomarkers exist and they underpin clinical decision-making. Nat Rev Clin Oncol.

[66] Abramson RG, Arlinghaus LR, Dula AN (2016). MR imaging biomarkers in oncology clinical trials. Magn Reson Imaging Clin N Am.

